# N^6^ ‐Methyladenosine Demethylase FTO Protects Pancreatic ΒTC6 Cells From Cholesterol‐Triggered Damage by Regulating *Nfatc4*: Insights From RNA‐Seq Analysis

**DOI:** 10.1111/cbdd.70413

**Published:** 2026-10-04

**Authors:** Pingping Hong, Yongjie Li, Jiandong Lin, Lingxiao Wang, Xiaofang Fan, Hongfang Wang, Luping Lou

**Affiliations:** ^1^ Department of Endocrinology Shaoxing Central Hospital Shaoxing Zhejiang China; ^2^ Department of Laboratory Shaoxing Central Hospital Shaoxing Zhejiang China

**Keywords:** ΒTC6, FTO, lipotoxicity, N^6^‐methyladenosine modification, pyroptosis, RNA‐seq analysis

## Abstract

Excessive cholesterol contributes to pancreatic β‐cell dysfunction and plays a critical role in the pathogenesis of metabolic disorders. FTO is a well‐characterized N^6^‐methyladenosine (m^6^A) demethylase involved in the regulation of RNA metabolism; however, its role in pancreatic β‐cells under cholesterol‐induced stress remains poorly understood. The effects of cholesterol on cell viability, apoptosis, pyroptosis, and FTO expression were examined in murine pancreatic BTC6 cells. The functional significance of the FTO–NFATC4 axis was investigated through *Fto* and *Nfatc4* overexpression. RNA sequencing, mRNA stability assays, luciferase reporter assays, and subcellular fractionation analyses were performed to elucidate the underlying regulatory mechanisms. Cholesterol treatment significantly reduced cell viability and promoted apoptosis and pyroptosis in BTC6 cells, accompanied by marked downregulation of FTO expression. RNA‐seq analysis identified *Nfatc4* as a downstream target of FTO. Mechanistically, *Fto* overexpression accelerated *Nfatc4* mRNA degradation and directly regulated the *Nfatc4* 3′‐UTR, resulting in decreased *Nfatc4* expression. Furthermore, *Fto* overexpression reduced the nuclear accumulation of NFATC4 in cholesterol‐treated BTC6 cells. Functionally, forced *Nfatc4* expression largely reversed the protective effects of FTO, leading to increased apoptosis and pyroptosis as well as reduced cell viability. FTO protects BTC6 cells against cholesterol‐induced injury by suppressing *Nfatc4* expression and limiting NFATC4 nuclear accumulation. These findings identify a novel FTO‐NFATC4 regulatory axis in pancreatic β‐cells and provide new insights into the molecular mechanisms underlying cholesterol‐induced β‐cell dysfunction, highlighting this pathway as a promising molecular target for the intervention of lipotoxic‐mediated pancreatic islet injury.

## Graphical abstract

FTO Protects Mouse Pancreatic Beta‐TC‐6 cells From Cholesterol‐Triggered Damage by Repressing *Nfatc4*.

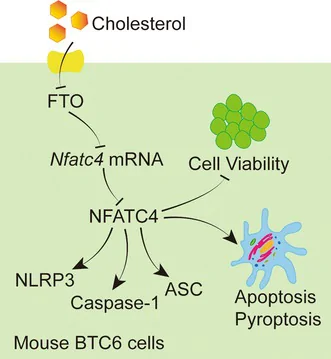

**Abbreviations**
ActD: actinomycin D
DEGs: differentially expressed genes
EMT: epithelial‐mesenchymal transition
FTO: fat mass and obesity‐associated protein
m^6^A: N^6^‐methyladenosine
qPCR: quantitative PCR
RT: room temperature
SD: standard deviation

## 1 Introduction

Excess cholesterol can damage pancreatic cells, leading to disorders like pancreatitis and diabetes (Perego et al. 2019). It accumulates in insulin‐producing β cells, causing inflammatory and oxidative stress, which impair insulin production and release (Fryirs et al. 2009; Manandhar et al. 2024). This is particularly relevant in Type 2 diabetes, where insulin resistance requires increased insulin production (Li et al. 2022). Over time, cholesterol‐induced damage can cause beta‐cell death, worsening hyperglycemia and advancing diabetes (Galli et al. 2023).

N^6^‐methyladenosine (m^6^A) is a specific adenosine methylation found in eukaryotic mRNA (Xu et al. 2022). It is a dynamic and reversible process modulated by m^6^A writers (methyltransferases), erasers (demethylases), and readers (binding proteins) (Yang et al. 2018; Kansal et al. 2026). m^6^A controls a broad range of biological processes, including those related to pancreatic cell dysfunction (Xia et al. 2019; Li et al. 2021). Among the regulators of m^6^A, fat mass and obesity‐associated protein (FTO) was initially considered a demethylase, and it has been linked to cell survival and viability (Shen et al. 2022). FTO could enhance the proliferation of different cell types (Deng et al. 2021; Huang et al. 2023) and inhibit programmed cell death like apoptosis and pyroptosis (Lin et al. 2023; Shen et al. 2021; Sun et al. 2023; Tu et al. 2024). In the context of pancreatic disorders, FTO is particularly significant; it facilitates pancreatic cancer development by enhancing PDGFC activity (Tan et al. 2022), and targeting FTO may hinder pancreatic cancer progression by diminishing the stemness and epithelial‐mesenchymal transition (EMT) of cancer cells (Garg et al. 2022).

FTO exhibits prominent cell‐context‐dependent functions in the pancreas. In pancreatic cancer cells, FTO is highly expressed and exerts pro‐survival effects; *FTO* knockdown impairs cell proliferation and promotes apoptosis (Tang et al. 2019). In contrast, in normal pancreatic islet β‐cells, high FTO expression has been reported to be associated with β‐cell dysfunction: *FTO* overexpression suppresses insulin secretion, triggers ROS production, and activates the NF‐κB signaling pathway (Fan et al. 2015). These seemingly contradictory functional differences suggest that the biological roles of FTO are highly dependent on specific cell types and stress microenvironments. Although the oncogenic function of FTO in pancreatic cancer has been extensively investigated, FTO's specific role in pancreatic cell dysfunction, especially related to excessive cholesterol and the corresponding mechanisms, remains incompletely elucidated.

To address these gaps, we exposed mouse pancreatic ΒTC6 cells to cholesterol, which caused fewer viable cells and increased apoptosis. Elevated cholesterol levels significantly lowered FTO expression, while overexpressing *Fto* alleviated pyroptosis and apoptosis and increased the number of viable cholesterol‐treated ΒTC6 cells. We then conducted RNA‐seq analysis, which revealed that *Nfatc4* was a significantly downregulated gene in *Fto*‐overexpressing cholesterol‐treated ΒTC6 cells. FTO suppressed *Nfatc4* transcription and altered its protein subcellular distribution, and overexpressing *Nfatc4* countered FTO's protection of these cells. Our results underscore the crucial function of the FTO‐NFATC4 axis in mediating cholesterol‐induced damage to pancreatic beta cells.

## 2 Materials and Methods

### 2.1 Cell Culture

ΒTC6 cells (#IM‐M053, IMMOCELL, Xiamen, China) were maintained with DMEM medium containing 15% fetal bovine serum (#A5670701, Gibco, Waltham, USA), streptomycin (100 μg/mL)/penicillin (100 U/mL) mixture (#15140148, Gibco) in a designated incubator set at 37°C and supplemented with 5% CO_2_.

### 2.2 Plasmids

The *Fto* or *Nfatc4* coding sequences were cloned into the pCDH‐EF1‐Flag‐T2A‐Puro or pcDNA3.1–3× Flag plasmids, resulting in constructs referred to as *Fto* OE and *Nfatc4* OE, respectively. The wild‐type or mutant 3′UTR of mouse *Nfact4* was inserted into the pGL3 vector. Cell transfections were done by the ExFect Transfection reagent (#T101‐01, Vazyme, Nanjing, China), according to the manufacturer's protocol. All plasmids underwent sequencing prior to transfection to ensure accuracy. The primers are as follows: *Fto* OE‐Forward: 5′‐TAGAGCTAGCGAATTCGCCACCATGGATTACAAGGACGACGATGAC‐3′, *Fto* OE‐Reverse: 5′‐TCAGCGGCCGCGGATCCGGGTTTTGCTTCCAGAAGCTG‐3′; *Nfatc4* OE‐Forward: 5′‐TACCGAGCTCGGATCCGCCACCATGGGGGCCGCAAGCTGCGAG‐3′, *Nfatc4* OE‐Reverse: 5′‐GCCCTCTAGACTCGAGGGCAGGAGGCTCTTCTCCAGG‐3′. *Nfatc4* 3′UTR‐Wt‐F: 5′‐GCTCGCTAGCCTCGAGACTGAGGCCCAGTGGAATAG‐3′, *Nfatc4* 3′UTR‐Wt‐R: 5′‐ATGCCTGCAGGTCGACCAAGGCAGCACCAGGCCTG‐3′; *Nfatc4* 3′UTR‐Mut‐F: 5′‐AACCACGTACTAGGTCATCACCTGGCAACCCCAG‐3′, *Nfatc4* 3′UTR‐Mut‐R: 5′‐TGATGACCTAGTACGTGGTTCAGGCAGGAGG‐3′.

### 2.3 CCK‐8 Assay

Cell viability was determined by a CCK‐8 assay kit (#G021‐1‐1, Njjcbio, Nanjing, China) as per the manufacturer's manual. Cells were initially plated into a 96‐well plate (2000 cells per well). After culturing for 12 h, the cells were exposed to varying cholesterol concentrations for 24 h or transfected with 0.2 μg of the designated plasmids and then cultured for 24 h in the presence of cholesterol at its IC_50_ concentration. Following this, the cells were exposed to 10 μL of CCK‐8 for 1 h at room temperature (RT), protected from light. The OD_450_ was then measured by a microplate reader. Each experiment involved technical triplicate and was repeated independently three times.

### 2.4 Apoptosis

Apoptotic cell death was assayed via a kit from Vazyme (#A211‐01, China), in accordance with the manufacturer's manual. Cells were inoculated into 6‐well plates (1 × 10^6^ cells/well) and maintained with or without 502.5 μM of cholesterol for 24 h. Alternatively, cells were plated into 96‐well plates (2000 cells/well), delivered with control, *Fto* OE, or *Nfatc4* OE plasmids, and then cultured with 502.5 μM of cholesterol for another 24 h. Eventually, the cells were incubated with Annexin V‐FITC/PI solutions before being assayed via flow cytometry using a NovoCyte Flow Cytometer (Agilent, Santa Clara, USA) along with its corresponding software.

### 2.5 RNA‐Seq and Bioinformatic Analysis

Purified RNA (1 μg) from each sample was analyzed using the Bioanalyzer 2100 system (Agilent) and then processed for library preparation using the NEBNext Ultra Kit for Illumina (#E7770, New England Biolabs, Ipswich, USA), based on the manufacturer's instructions. The library was processed through the Illumina platform with 150 bp paired‐end reads. The raw sequence data were filtered and aligned to GRCm38 using Hisat2 (v2.0.5). StringTie (v1.3.3b) was employed to assemble the mapped reads using a reference‐based approach. The number of mapped reads to each gene was counted by FeatureCounts (v1.5.0‐p3), and gene expression levels were calculated as FPKM. Differentially expressed gene (DEG) identification was conducted using DESeq2 (v1.20.0), with criteria of |Log2FoldChange| > 0 and a *p*‐value < 0.05. Enrichment analysis for DEGs was performed using clusterProfiler (v4.0) with reference to KEGG pathways. *padj* < 0.05 was considered statistically significant. Data presentation, including heatmaps and bar plots, was generated using “pheatmap” (v1.0.12) and “ggplot2” (v3.5.1), respectively, in R (v4.3.0).

### 2.6 Quantitative PCR (qPCR)

RNA isolater reagent (#R401‐01, Vazyme) was utilized to isolate and purify total RNA, which was then used for cDNA synthesis using the HiScript II Q RT SuperMix (#R223‐01, Vazyme). qPCR was done using ChamQ SYBR qPCR Master Mix (#Q311‐01, Vazyme) on an ABI 7300 Plus system (BioRad, Hercules, USA). Specifically, add 1 mL of RNA isolater to the cells and dislodge the cells by pipetting. Transfer the lysate to a centrifuge tube and repeatedly pipette the mixture until no obvious particles are observed. Allow the lysate to stand on ice for 5 min. Add chloroform at 1/5 of the lysate volume, shake vigorously for 15 s, and incubate at 4 C for 5 min. Centrifuge at 12000 g for 15 min at 4°C. Carefully transfer the upper aqueous phase to a new centrifuge tube. Add an equal volume of isopropanol and mix thoroughly by inverting the tube. Place the sample at −20 C for 30 min. Centrifuge at 12000 g for 10 min at 4 C. Dissolve the RNA pellet with DEPC‐treated water, and measure the RNA concentration and the A_2_₆₀/A_2_₈₀ ratio. Reverse‐transcription reaction conditions: 25 C for 10 min; 50 C for 30 min; 85 C for 5 min. qPCR thermal cycling program: pre‐denaturation (95 C, 30 s); amplification cycles (95 C, 10 s; 60 C, 30 s); melting‐curve analysis (95 C, 15 s; 60 C, 60 s; 95 C, 15 s). The relative levels of the target transcripts were confirmed based on the 2^−ΔΔCt^ method. qPCR primers are listed as follows: q*Fto*‐F: 5′‐CCGTCCTGCGATGATGAAGT‐3′, q*Fto*‐R: 5′‐CCCATGCCGAAATAGGGCTC‐3′; q*Nfatc4*‐F: 5′‐GAGCTGGAATTTAAGCTGGTGT‐3′, q*Nfatc4*‐R: 5′‐CATGGAGGGGTATCCTCTGAG‐3′; 18S rRNA‐F: 5′‐CGACGACCCATTCGAACGTCT‐3′, 18S rRNA‐R: 5′‐CTCTCCGGAATCGAA CCCTGA‐3′.

### 2.7 Western Blot

Protein was extracted using RIPA buffer with added phosphatase inhibitors (Beyotime, Shanghai, China) through homogenization on ice. For isolation of nuclear and cytoplasmic fractions, a kit from Beyotime was applied (P0027). The concentrations of the extracted proteins were measured using a BCA kit from TIANGEN (#PA115, Beijing, China). Proteins (20 μg) were then separated via 10% SDS‐PAGE and transferred onto PVDF membranes, which were blocked with 3% BSA for 1 h at RT, followed by overnight incubation with appropriate antibody solutions at 4°C. The next day, the membranes were incubated with secondary antibodies for 1 h at RT after washing with TBST (TBS + 0.1% Tween 20). Protein bands were detected using an ECL kit (#P0018M, Beyotime, China) and visualized by exposure to X‐ray films. Bands were analyzed using ImageJ. The antibodies used are listed as follows: NFATC4 (#26311‐1‐AP, Proteintech, 1:500, Wuhan, China), FTO (#27226‐1‐AP, Proteintech, 1:500), ASC (#A43032203, AdipoGen, 1:500, San Diego, USA), Pro‐Caspase 1 (#ab179515, Abcam, 1:500, Cambridg, UK), NLRP3 (#19771‐1‐AP, Proteintech, 1:500), Cleaved‐Caspase 1 (C‐caspase 1, #RLC0003, Immunoway, 1:2000, San Jos, USA), Histone H3 (#M1309, HUABio, 1:500), GAPDH (#AC033, Abclonal, 1:1000), β‐actin (#20536‐1‐AP, Proteintech, 1:1000), HRP‐Anti‐Mouse IgG (#SA00001‐1, Proteintech, 1:10000) and HRP‐Anti‐Rabbit IgG (#SA00001‐2, Proteintech, 1:10000).

### 2.8 Luciferase Reporter Assay

The Duo‐Lite Luciferase Assay System (DD1205‐01, Vazyme) was applied for the measurement of luciferase activity. Briefly, BTC6 cells were seeded into 96‐well plates; each well was transfected with 0.4 μg of reporter plasmids and 0.4 μg of *Fto* OE. After 48 h, the cells were rinsed with PBS and subjected to luciferase activity assessment following the manufacturer's protocol using a Promega Luminometer. Firefly luciferase activity for each well was calibrated with Renilla luciferase activity.

### 2.9 Statistical Analysis

GraphPad Prism (v9) was utilized for all statistical analyses. For comparisons between two data groups, an unpaired two‐sided *t*‐test was applied to assess the significance of parametric data. When comparing multiple groups of data, a one‐way ANOVA together with Tukey's HSD post hoc test was employed. Results are displayed as the mean ± standard deviation. *p*‐values < 0.05 were set as the statistical significance threshold. All the data were independently replicated biologically three times, with CCK‐8, qPCR, and luciferase reporter assays additionally conducted in technical triplicates.

## 3 Results

### 3.1 Cholesterol Inhibits the Expression of FTO in Pancreatic ΒTC6 Cells

To confirm cholesterol's influence on pancreatic secretory cells, we treated mouse pancreatic ΒTC6 cells with graded doses of cholesterol. CCK‐8 assay data revealed that cholesterol reduced cell viability dose‐dependently, and the IC_50_ of cholesterol is 502.5 μM (Figure 1A). Consistently, cholesterol at its IC_50_ significantly induced the apoptotic cell death of ΒTC6 cells (Figure 1B,C).

**FIGURE 1:**
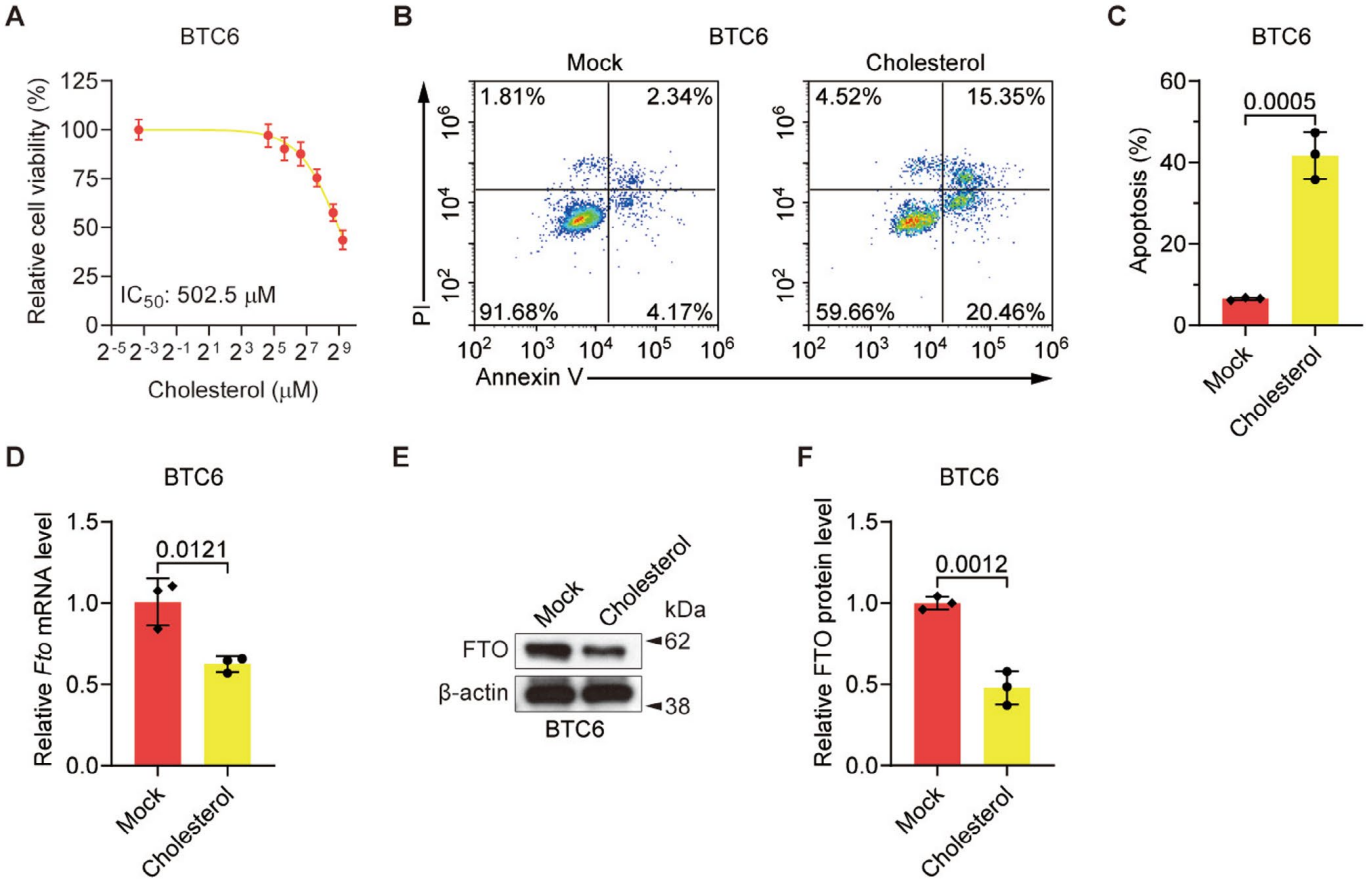
Cholesterol represses FTO expression in ΒTC6 cells. (A) Results of CCK‐8 assay showed ΒTC6 cell viability in the presence of indicated cholesterol concentrations. (B, C) Annexin V/PI staining results showed the apoptotic cell death of control and cholesterol‐induced ΒTC6 cells. Mock defines treatment of the same volume of solvent as cholesterol. (D) qPCR data illustrating the mRNA levels of *Fto* in control and cholesterol‐treated ΒTC6 cells. (E, F) Western blot indicated the reduced protein level in cholesterol‐treated cells compared to control cells. The values in the statistical plots are *p* values. *p*‐values were calculated by unpaired Student's *t*‐test.

To find out whether cholesterol affects FTO expression in these cells, we performed qPCR and Western blot assays. The data showed that FTO expression is markedly decreased in cholesterol‐induced ΒTC6 cells (Figure 1D–F). These results suggest FTO's potential function in cholesterol‐treated ΒTC6 cells.

### 3.2 *Fto* Overexpression Attenuates Cholesterol‐Triggered Damage to ΒTC6 Cells

To study the function of FTO in cholesterol‐induced ΒTC6 cells, we overexpressed *Fto* in these cells. qPCR and Western blot confirmed its successful overexpression (Figure 2A–C). Given cholesterol's function in regulating inflammation, viability, and cell death of ΒTC6 cells (Hong et al. 2023), we initially evaluated the expression of markers related to pyroptosis, a regulated programmed cell death highly associated with inflammation (Hsu et al. 2021; Hou et al. 2021). Western blot revealed that the increased expression of pyroptosis‐associated markers induced by cholesterol, including NLRP3, ASC, and Cleaved Caspase 1 (C‐caspase 1), was significantly inhibited upon *Fto* overexpression in ΒTC6 cells (Figure 2B,C). Consistent with this, *Fto* overexpression also mitigated the pro‐apoptotic effect of cholesterol in ΒTC6 cells, as indicated by Annexin V/PI staining data (Figure 2D,E). Moreover, CCK‐8 experiments uncovered that the impaired viability of ΒTC6 cells caused by cholesterol treatment was improved by excessive FTO (Figure 2F). Together, these observations suggest that FTO could protect ΒTC6 cells against cholesterol‐induced damage.

**FIGURE 2:**
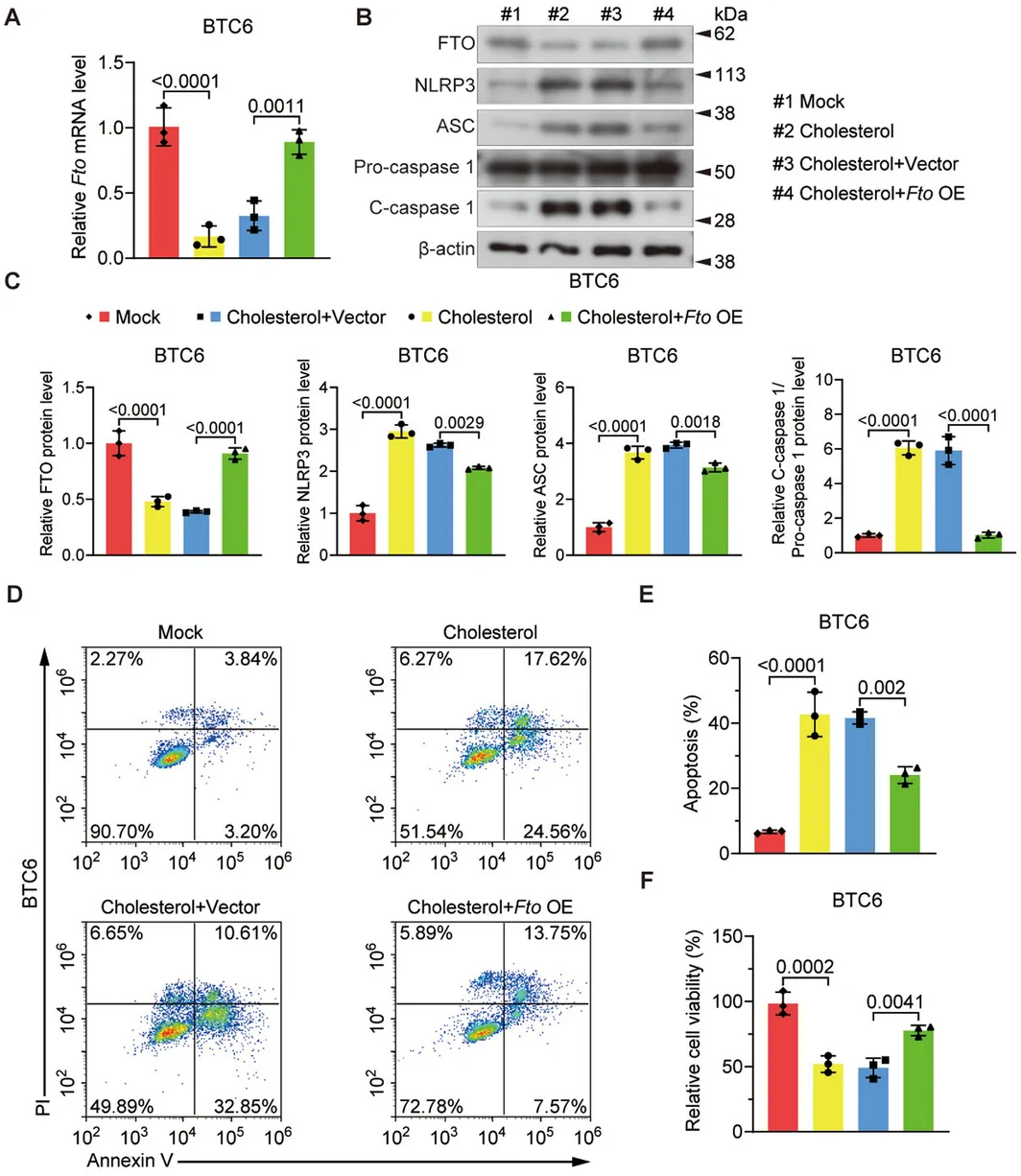
FTO protects ΒTC6 cells from cholesterol‐triggered pyroptosis, apoptosis, and viability impairment. (A) qPCR data showed the *Fto* mRNA levels in ΒTC6 with the indicated conditions. (B, C) Western blot results indicated the protein expression of FTO, NLRP3, ASC, Pro‐Caspase 1, and C‐caspase 1 in different groups. (D, E) Annexin V/PI staining results depicted the apoptotic cell death of different groups. (F) CCK‐8 staining data illustrating the viability of different groups. The values in the statistical plots are *p* values. *p*‐values were calculated by one‐way ANOVA followed by Tukey's honestly significant difference (HSD).

### 3.3 Transcriptomic Analysis of the Impact of *Fto* Overexpression on ΒTC6 Cells Treated With Cholesterol

To explain how FTO fulfills its function in ΒTC6 cells exposed to cholesterol, we performed RNA‐seq using RNAs extracted from cholesterol‐induced cells transfected with control or *Fto* OE plasmids. The results uncovered 103 upregulated and 169 downregulated genes in cells overexpressing *Fto* compared with control cells with cholesterol treatment (Figure 3A). Upregulated and downregulated top 10 genes with fold change are displayed in Figure 3B. These downregulated DEGs are associated with multiple KEGG pathways regulating cell survival, proliferation, and inflammation, such as the MAPK (Moens et al. 2013) and Wnt (Jridi et al. 2020) signaling pathways (Figure 3C). Among downregulated DEGs that are linked to these pathways, we paid attention to *Nfatc4*, which is involved in multiple pathways (Figure 3D). Therefore, we subsequently explored the role of NFATC4 in cholesterol‐induced *Fto*‐overexpressing ΒTC6 cells.

**FIGURE 3:**
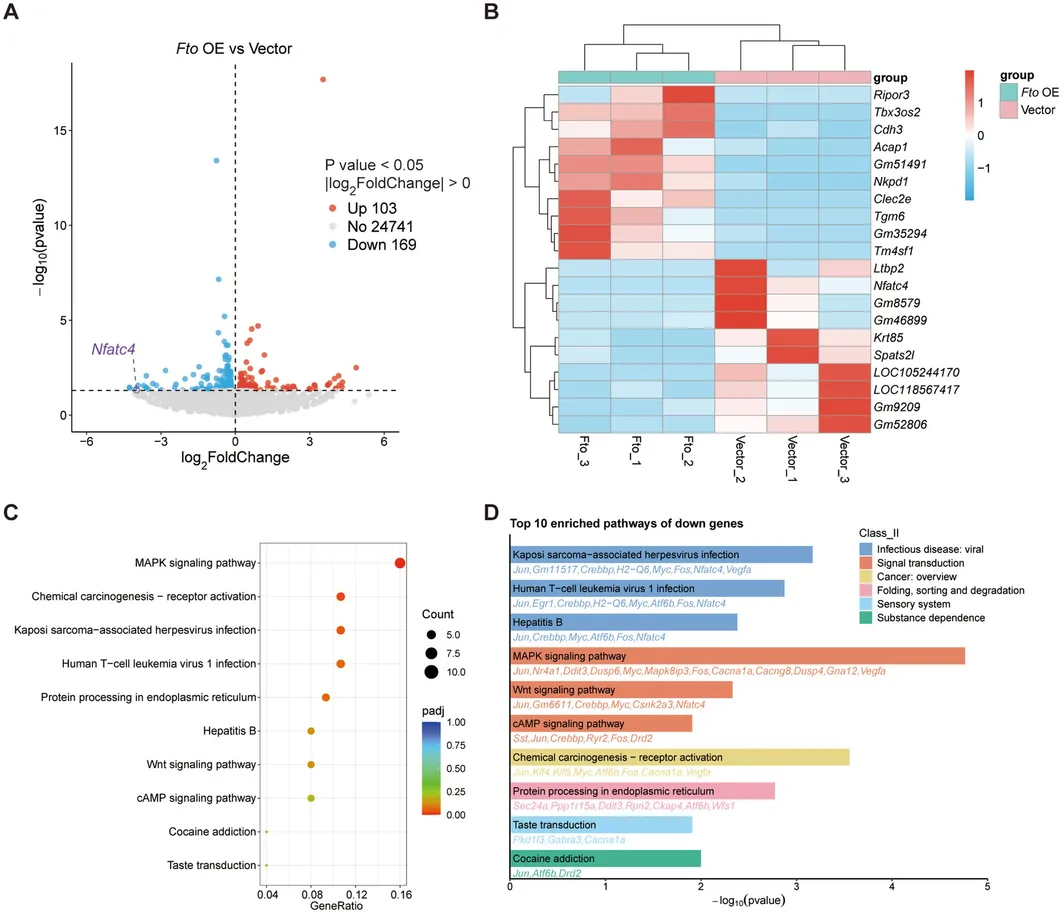
RNA‐seq of control and *Fto*‐overexpressing ΒTC6 cells exposed to cholesterol. (A) Volcano plot displayed DEG distribution between *Fto*‐overexpressing and control cells. (B) Heatmap depicted the levels of the top 10‐fold change upregulated and downregulated DEGs between *Fto*‐overexpressing and control cells. (C) KEGG pathways associated with downregulated DEGs. (D) Bar plot indicated the downregulated DEGs associated with the identified KEGG pathways.

### 3.4 FTO Negatively Regulates the Expression and Subcellular Distribution of NFATC4 in Cholesterol‐Induced ΒTC6 Cells

To determine the FTO's impact on NFATC4 expression in ΒTC6 cells exposed to cholesterol, we conducted qPCR and Western blot assays. The data revealed that NFATC4 abundance was substantially upregulated by cholesterol in these cells. However, excessive FTO significantly mitigated the elevated NFATC4 levels in these cells (Figure 4A–C). Since FTO is a well‐recognized mRNA stability regulator, we induced these cells with Actinomycin D (ActD) and evaluated *Nfatc4* mRNA degradation. qPCR data showed that *Fto* overexpression significantly accelerated *Nfatc4* mRNA degradation in cells treated with cholesterol (Figure 4D).

**FIGURE 4:**
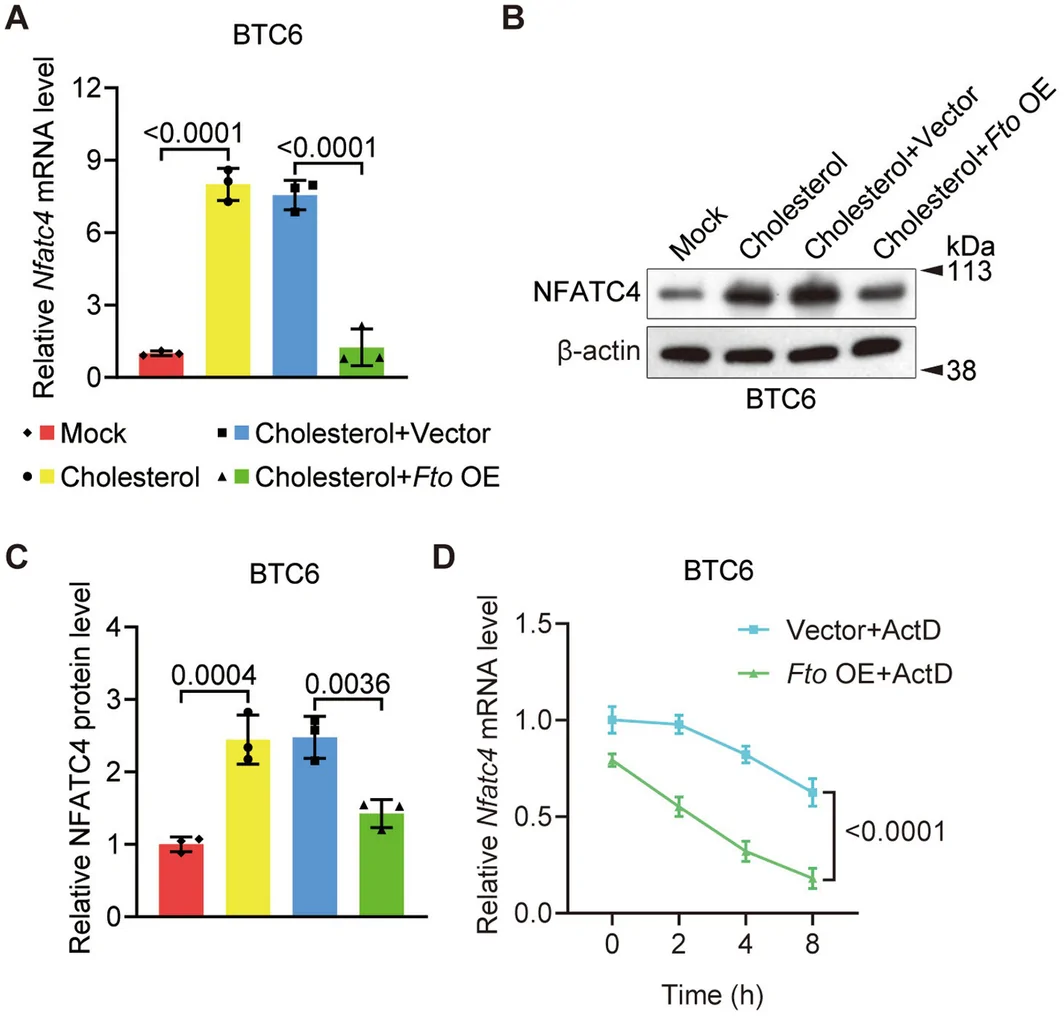
FTO inhibits NFATC4 expression in cholesterol‐induced ΒTC6 cells. (A) qPCR data showed *Nfatc4* mRNA level in different groups. (B and C) Western blot indicated NFATC4 protein abundance in different ΒTC6 cell groups. (D) qPCR data illustrated the levels of *Nfatc4* mRNA in control or *Fto*‐overexpressing ΒTC6 cells treated with 5 μg/mL ActD for the indicated time duration. The values in the statistical plots are *p* values. *p*‐values were calculated by one‐way ANOVA followed by Tukey's HSD in (A, C). Two‐way ANOVA followed by Sidak's multiple‐comparisons test was used for (D).

To find out whether FTO directly regulates *Nfatc4* transcripts, a luciferase reporter assay was conducted using constructs containing either the wild‐type (Wt) or mutant (Mut) *Nfatc4* 3′‐UTR, in which the predicted FTO‐binding site was disrupted. The results showed that *Fto* overexpression significantly altered the luciferase activity of the reporter containing the Wt *Nfatc4* 3′‐UTR, whereas no significant effect was observed with the mutant construct (Figure 5A), suggesting a direct interaction between FTO and *Nfatc4* mRNA. Furthermore, subcellular fractionation analysis demonstrated that *Fto* overexpression significantly reduced the nuclear accumulation of NFATC4 protein in cholesterol‐treated BTC6 cells. In contrast, the cytoplasmic level of NFATC4 remained largely unchanged following *Fto* overexpression (Figure 5B–E). Collectively, these findings indicate that FTO suppresses *Nfatc4* expression by promoting mRNA degradation and modulates the subcellular distribution of NFATC4 in BTC6 cells under cholesterol‐induced stress.

**FIGURE 5:**
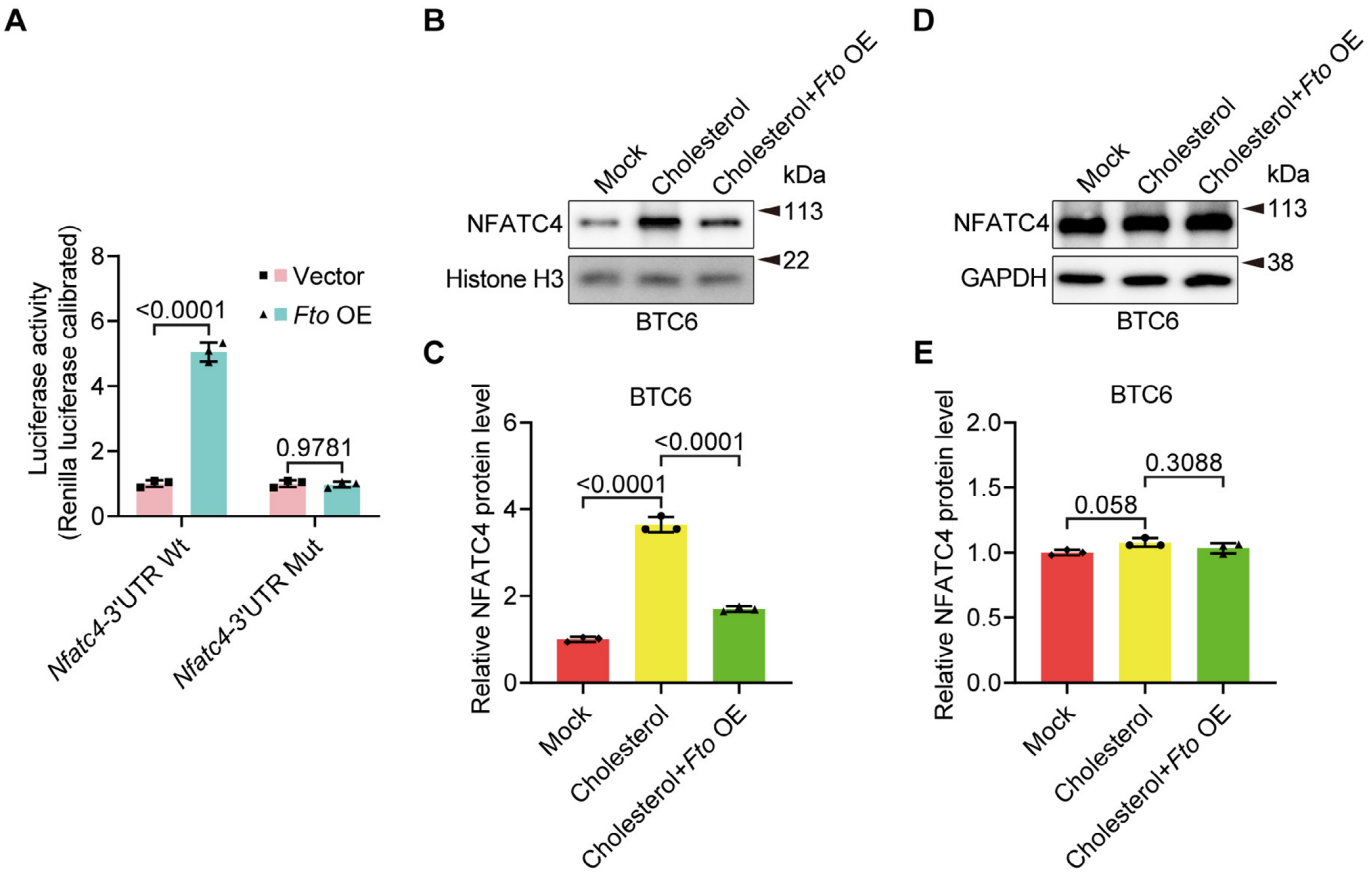
FTO directly regulates *Nfatc4* and suppresses nuclear accumulation of NFATC4 in cholesterol‐treated BTC6 cells. (A) Luciferase reporter assay showing the effect of *Fto* overexpression on the activity of reporters containing the Wt or Mut *Nfatc4* 3′‐UTR in BTC6 cells. (B‐E) Representative Western blot images showing NFATC4 protein levels in nuclear and cytoplasmic fractions of BTC6 cells treated with cholesterol and transfected with the indicated plasmids. Histone H3 and GAPDH were used as nuclear and cytoplasmic loading controls, respectively. Two‐way ANOVA followed by Sidak's multiple‐comparisons test was used for (A). *p*‐values were calculated by one‐way ANOVA followed by Tukey's HSD in (C, E).

### 3.5 Overexpression of *Nfatc4* Attenuates FTO's Protection on Cholesterol‐Induced ΒTC6 Cells

To directly test the function of NFATC4 in cholesterol‐induced *Fto*‐overexpressing ΒTC6 cells, we overexpressed *Nfatc4* in these cells. qPCR and Western blot data confirmed that *Nfatc4* was successfully overexpressed, but the level of FTO was unaltered in the presence of NFATC4 (Figure 6A–C). Notably, Western blot data showed that FTO's inhibition of NLRP3, ASC, and C‐caspase 1 protein levels was attenuated by excessive NFATC4, as the levels of these proteins were significantly upregulated in *Nfatc4/Fto‐overexpressing* cells (Figure 6B,C), indicating that pyroptosis was strengthened. Similarly, the apoptosis of *Fto*‐overexpressing cells was enhanced, while their viability was impaired upon *Nfatc4* overexpression (Figure 6D–F). These findings demonstrate that *Nfatc4* attenuates FTO's influence on the survival and viability of cholesterol‐induced ΒTC6 cells.

**FIGURE 6:**
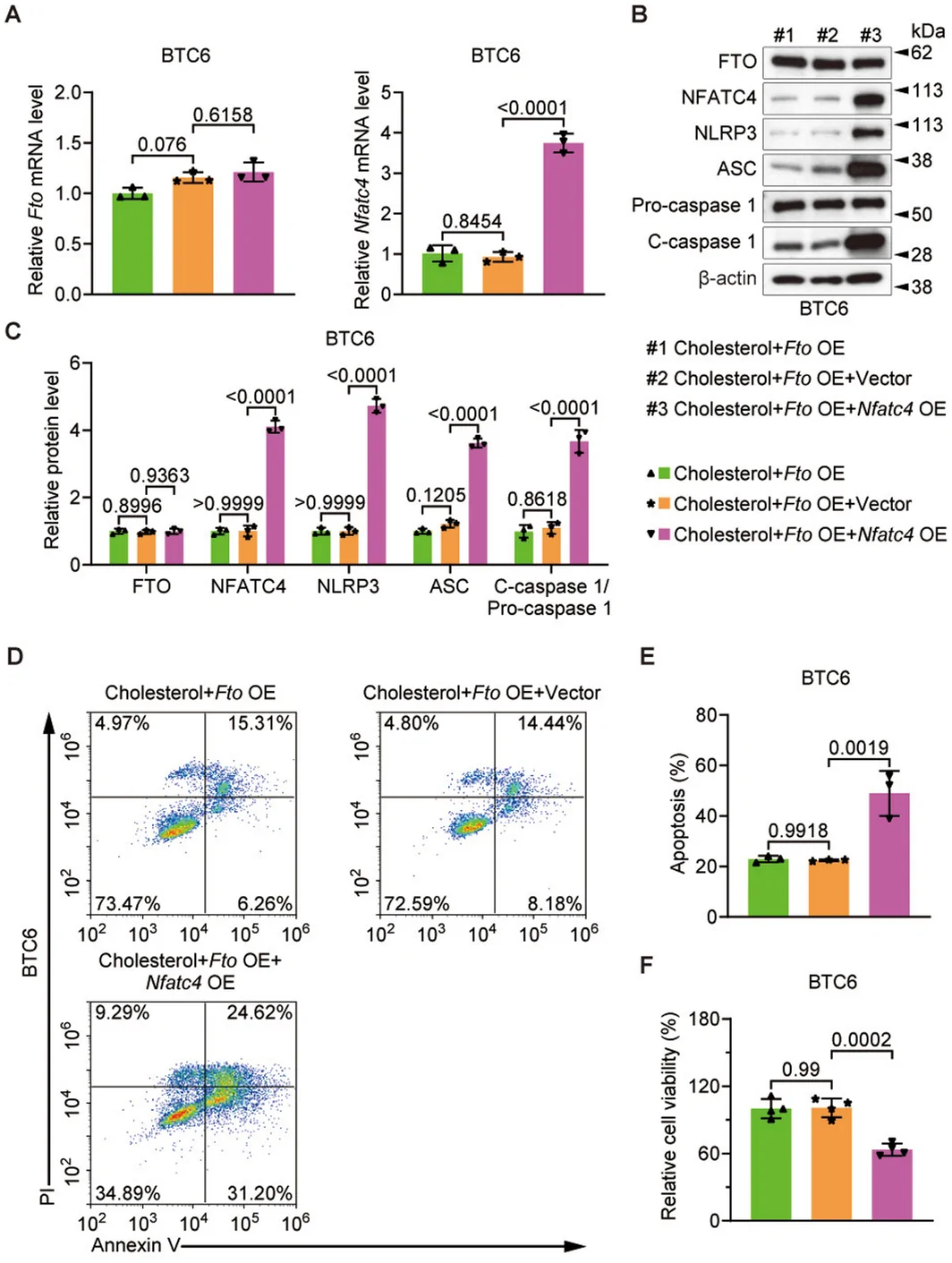
Excessive NFATC4 attenuates FTO's protective effect on the survival and viability of cholesterol‐treated ΒTC6 cells. (A) qPCR revealed *Fto* and *Nfatc4* mRNA levels in *Fto*‐overexpressing cholesterol‐induced ΒTC6 cells in the presence of or without *Nfatc4* overexpression. (B, C) Western blot indicated FTO, NFATC4, NLRP3, ASC, Pro‐Caspase 1, and C‐caspase 1 protein levels in *Fto*‐overexpressing cholesterol‐treated ΒTC6 cells in the presence of or without *Nfatc4* overexpression. (D, E) Annexin V/PI assays illustrated the apoptotic cell death of *Fto*‐overexpressing cholesterol‐treated ΒTC6 cells in the presence of or without *Nfatc4* overexpression. (F) CCK‐8 experiments depicted the viability of *Fto*‐overexpressing cholesterol‐treated ΒTC6 cells in the presence of or without *Nfatc4* overexpression. The values in the statistical plots are *p* values. *p*‐values were calculated by one‐way ANOVA followed by Tukey's HSD.

## 4 Discussion

Dysregulated m^6^A modification and abnormal expression or activity of m^6^A regulators have been linked to various diseases (Jiang et al. 2021). In pancreatic tissue, significant effort has been made to investigate m^6^A's regulation in pancreatic cancer (Liu et al. 2023; Hu et al. 2022). By contrast, its function in noncancerous pancreatic cells remains poorly understood. In this manuscript, we studied the function of m^6^A eraser FTO in cholesterol‐induced mouse pancreatic cells. Our data showed that FTO protects the ΒTC6 cells from cholesterol‐triggered pyroptosis and apoptosis and improves their viability.

In these cells, FTO levels are markedly reduced by cholesterol. On the other hand, increased FTO expression is observed in malignant pancreatic cells (Garg et al. 2022). How FTO expression is controlled in the context of pancreatic cells, and whether cholesterol and carcinogenesis of pancreatic cells employ a similar mechanism to regulate its expression remain to be determined.

The function of FTO is context‐dependent. For instance, FTO can facilitate pancreatic cancer progression (Tan et al. 2022), but it also functions to suppress tumorigenesis in other types of cancers (Zuidhof and Calkhoven 2022). This discrepancy is likely due to its diverse substrates in different scenarios (Zuidhof and Calkhoven 2022). Therefore, identifying its targets in a specific context is critical to delineate the mechanism by which it fulfills its function. Through RNA‐seq analysis, we identified *Nfatc4* as a significantly downregulated gene in cholesterol‐treated ΒTC6 cells that overexpress *Fto*.

NFATC4, also known as NFAT3, is a member of the NFAT family, which consists of five proteins (NFAT1‐5) (Müller and Rao 2010). These factors are known for their function in regulating the immune system (Vaeth and Feske 2018). However, their roles in other contexts, such as tumorigenesis, are also recognized (Pan et al. 2013). In pancreatic cells, the NFAT signaling promotes proliferation and strengthens insulin secretion (Heit et al. 2006), and the activated NFAT pathway also facilitates pancreatic cancer progression by promoting proliferation, survival, and migration of cancer cells (König et al. 2010). Unlike other NFATs, NFATC4 expression is lower in immune cells due to the lack of TBX5 expression, which is a key factor controlling its expression (Kaminuma et al. 2018). However, its multifaceted role in other scenarios has been implicated (Zhong et al. 2022). NFATC4 facilitates the self‐renewal and division of neural stem cells (Moreno et al. 2015). It also regulates acinar‐to‐ductal trans‐differentiation within pancreatic tissue and facilitates its tumorigenesis by inducing SOX9 (Hessmann et al. 2016). Interestingly, our finding uncovers NFATC4's pro‐pyroptotic, pro‐apoptotic, and anti‐viability functions in cholesterol‐induced ΒTC6 cells, indicating the context‐dependent regulation of NFATC4 in pancreatic cells. Future exploration should be planned to uncover the mechanism underlying NFATC4's influence on pancreatic cells' survival and viability.

The primary function of FTO is to modulate the stability of its client RNA. In support of this notion, our data show that *Fto* overexpression influences the mRNA level of *Nfatc4*, at least partially by promoting its degradation. Nevertheless, whether FTO directly removes the m^6^A modification of *Nfatc4* mRNA has not been examined. It is also possible that FTO controls other factors that affect the transcription of *Nfatc4*, thereby indirectly affecting its mRNA expression. Additionally, given its broad substrates, FTO may fulfill its protective function by regulating additional targets and mechanisms.

Interestingly, *Fto* overexpression markedly reduced nuclear NFATC4 levels while exerting minimal effects on the cytoplasmic fraction. One possible explanation is that FTO impairs NFATC4 nuclear translocation, resulting in the retention of NFATC4 within the cytoplasm. In this context, the reduction in NFATC4 synthesis caused by FTO‐mediated mRNA destabilization may be offset by the accumulation of NFATC4 that fails to enter the nucleus, thereby masking changes in the cytoplasmic pool. Consequently, the inhibitory effect of FTO is more evident in the nuclear compartment, where reduced NFATC4 accumulation is expected to attenuate its transcriptional activity. Further studies are needed to determine whether FTO directly regulates the nuclear trafficking of NFATC4.

Several limitations exist in the present study, and further investigations are warranted. First, all functional experiments were performed solely in BTC6 cells. Future work will repeat core assays in multiple pancreatic islet cell lines to verify the generalizability of our conclusions. Second, although downstream differentially expressed genes of FTO were screened via RNA‐seq, the exact m^6^A modification sites on *Nfatc4* and other candidate targets regulated by FTO remain uncharacterized. Subsequent m^6^A MeRIP‐seq will be applied to validate their m^6^A modification profiles and clarify the precise regulatory mechanisms. Third, this study is restricted to in vitro cellular experiments; in vivo animal models are required to confirm the protective effect of FTO against lipotoxic injury and pyroptosis in pancreatic islets. Furthermore, the upstream and downstream mechanisms by which FTO modulates NFATC4 nuclear translocation remain to be explored. Additional novel targets mined from our RNA‐seq‐derived differentially expressed genes will help to further delineate the molecular network underlying FTO‐mediated regulation of pancreatic lipotoxic injury.

## 5 Conclusion

To summarize, our data provide direct evidence that FTO protects pancreatic cells from cholesterol‐triggered damage, likely through repressing the *Nfatc4* expression. These observations deepen our understanding of FTO's role in pancreatic tissue and underscore the therapeutic potential of FTO targeting in treating conditions involving pancreatic cell dysfunction.

## Author Contributions

**Pingping Hong:** writing – original draft, data curation, methodology, formal analysis, funding acquisition, writing – review and editing. **Yongjie Li:** software, data curation, methodology, supervision, formal analysis. **Jiandong Lin:** supervision, validation, software. **Lingxiao Wang:** supervision, validation, visualization. **Xiaofang Fan:** methodology, supervision, formal analysis. **Hongfang Wang:** supervision, validation, visualization. **Luping Lou:** writing – review and editing, conceptualization, investigation, project administration, resources, visualization.

## Funding

This study was supported by The Medical Science and Technology Project of Zhejiang Province (Grant 2024KY1740, 2025HY1328).

## Ethics Statement

The authors have nothing to report.

## Consent

The authors have nothing to report.

## Conflicts of Interest

The authors declare no conflicts of interest.

## Data Availability

The authors confirm that the data supporting the findings of this study are available from the corresponding authors upon reasonable request.
